# Non-contact co-culture with human vascular endothelial cells promotes epithelial-to-mesenchymal transition of cervical cancer SiHa cells by activating the NOTCH1/LOX/SNAIL pathway

**DOI:** 10.1186/s11658-019-0163-z

**Published:** 2019-06-10

**Authors:** Jinghua Ou, Defeng Guan, Yongxiu Yang

**Affiliations:** 1 The First Clinical Medical College of Lanzhou University, NO 1 West Donggang Road, Chengguan District, Lanzhou, Gansu China; 2 Department of obstetrics and gynecology, Gansu provincial hospital, Lanzhou, Gansu China; 3 Department of Obstetrics and Gynecology, The First Hospital of Lanzhou University, NO 1 West Donggang Road, Chengguan District, Lanzhou, Gansu China

**Keywords:** Co-culture, Cervical cancer, SiHa cells, HUVECs, NOTCH/LOX/SNAIL pathway, EMT

## Abstract

**Background:**

The aim of this study was to investigate the effect of human umbilical vein endothelial cells on epithelial-to-mesenchymal transition of the cervical cancer cell line SiHa by studying the Notch1/lysyl oxidase (LOX)/SNAIL1 pathway.

**Methods:**

Monocultures of SiHa cells, SiHa cells containing a control sequence, and *Notch1*-silenced SiHa cells, as well as co-cultures of human umbilical vein endothelial cells with SiHa cells and *Notch1*-silenced SiHa cells, were established. The invasiveness of SiHa cells in each group was evaluated using a Transwell assay. The mRNA levels of E-cadherin and vimentin were detected using quantitative real-time polymerase chain reaction. The expression levels of the matrix metalloproteinases MMP-2 and MMP-9 were determined in SiHa cells using an immunofluorescence assay and the protein activity was detected by gelatin zymography. Changes in LOX, SNAIL1 and NOTCH1 expression in the SiHa cells in each group were detected using western blotting.

**Results:**

Compared with monocultured SiHa cells, co-cultured SiHa cells showed a significant increase in their invasiveness and expression levels of vimentin, as well as of NOTCH 1, LOX, and SNAIL1, whereas their expression of E-cadherin was significantly reduced and protein activities of MMP-2 and MMP-9 were increased. Compared with SiHa, mono- and co-cultured *NOTCH 1*-silenced SiHa cells showed significant reductions in their invasiveness and expression levels of vimentin, NOTCH 1, LOX, and SNAIL1, whereas their expression of E-cadherin significantly increased and protein activities of MMP-2 and MMP-9 decreased.

**Conclusion:**

Co-culture with human umbilical vein endothelial cells promoted the epithelial-to-mesenchymal transition of SiHa cells by activating the NOTCH1/LOX/SNAIL1 pathway in SiHa cells, which enhanced their invasive and metastatic capacities. The results of this study may provide a new perspective on cervical cancer metastasis and a theoretical basis for clinical treatment.

## Background

Cervical cancer is the fourth most common type of cancer in women worldwide, with an estimated 530,000 new cases each year. It is one of the leading causes of cancer-related deaths in women, with an estimated 270,000 deaths annually [1]. Approximately 85% of cervical cancer deaths in the world occur in less developed or developing countries [2]. In China, the incidence and rate of mortality from cervical cancer continue to rise; for example, in 2015 alone, the number of new cervical cancer cases reached 98,900, thus accounting for 18.7% of the global incidence and becoming a major public health issue in China [3]. Studies have found that cervical cancer is more prone to metastasis than other types of cancer, and is one of the leading causes of death in patients with cervical cancer. Therefore, inhibiting or delaying metastasis of cervical cancer cells is of great significance in prolonging survival and improving the quality of life of patients. Cervical cancer metastasis is closely related to epithelial-to-mesenchymal transition (EMT), whereby epithelial tumour cells acquire a mesenchymal phenotype, which allows for the invasion and metastasis of tumour cells [4]. Studies have found that abnormally elevated levels of multiple factors, including long noncoding RNAs, microRNAs, and transforming growth factor (TGF)-β, as well as signalling pathways such as the nuclear factor-κB, WNT, and NOTCH pathways, can activate EMT and promote metastasis in cervical cancer [5–10].

In tumour tissues, tumour and non-tumour cells interact to promote tumour development. Cancer progression is closely associated with the tumour microenvironment, including fibroblasts, immune cells, endothelial cells, blood vessels and proteins produced [11]. Among non-tumour cells, inflammatory cells are implicated in the persistent proliferation and immunosuppression-mediated escape of tumour cells [12]. In addition, the hypoxia-induced migration of endothelial cells and angiogenesis play important roles in promoting tumour growth, metastasis, and progression [13]. EMT confers characteristics of mesenchymal cells to tumour cells, which then exhibit high motility and can easily enter the bloodstream by degrading tumour tissues and blood vessel walls, resulting in metastasis. Non-contact culture can make tumour cells interact with other cells through paracrine factors, providing a microenvironment for tumour cells, and allowing further studies of the formation, occurrence and development of cancer, as well as the treatment mechanism [11].

Interactions between tumour cells and vascular endothelial cells have been shown to promote tumour cell metastasis in multiple tumour types [14, 15]. Previous studies have mainly focused on the effects of tumours on blood vessel formation [16]. However, considering cell-cell interactions and the bidirectionality of signal transduction, it is necessary to evaluate whether vascular endothelial cells can induce EMT of cervical cancer cells and promote tumour cell metastasis. Thus, in this study, we utilised non-contact co-culture of human vascular endothelial cells and the cervical cancer cell line SiHa to investigate the potential role and molecular mechanisms of normal human vascular endothelial cells in cervical cancer metastasis.

## Methods

### Cells and reagents

HEK293 cells and the human cervical cancer cell line SiHa were purchased from the Cell Bank of Type Culture Collection of the Chinese Academy of Sciences. Human umbilical vein endothelial cells (HUVECs) were purchased from the American Type Culture Collection. Two recombinant adenoviruses, Ad-control, with an empty capsid containing a control sequence, and Ad-Not-siRNA, containing a gene encoding a *NOTCH 1*-specific small interfering RNA (siRNA), were constructed by Sangon Biotech Co., Ltd. (Shanghai, China). High-glucose Dulbecco’s modified Eagle’s medium (DMEM) was purchased from Gibco, Thermo Fisher Scientific (Waltham, MA, USA). Foetal bovine serum (FBS) was purchased from Beijing Ever Green Biotechnology Co., Ltd. Trypsin, RIPA lysis buffer, hypersensitive enhanced chemiluminescence (ECL) detection reagents, sodium dodecyl sulphate polyacrylamide gel electrophoresis (SDS-PAGE) reagents, and western blotting membranes were purchased from Beyotime Biotechnology Co., Ltd. (Shanghai, China). Rabbit monoclonal antibodies against matrix metalloproteinase (MMP)-2, MMP-9, NOTCH 1, and SNAIL1 were purchased from Abcam (Cambridge, UK). DyLight 594-labelled goat anti-rabbit IgG was purchased from GeneTex (Irvine, CA, USA). Horseradish peroxidase-labelled goat anti-rabbit IgG was purchased from Cell Signaling Technology. Trizol, a reverse transcription kit, and real-time quantitative PCR kit were purchased from TaKaRa (Tokyo, Japan). Primers were synthesised by TaKaRa, and the primer sequences are shown in Table 1.

**Table 1:** Primer sequences

E-cadherin:	Upstream	5′-CCTACGATTTCCCATCACCA-3′
	Downstream	5′-GTCCACGGTCTCCTGTCTGT-3′
Vimentin	Upstream	5′-GGATTTCTCTGCCTCTTCCA-3′
	Downstream	5′-CACCTGTCCGTCTCTGGTTT-3′
β-actin	Upstream	5′-AGGGAAATCGTGCGTGACAT-3′
	Downstream	5′-GAACCGCTCATTGCCGATAG-3′

### Cell culture

Both SiHa cells and HUVECs were cultured in high-glucose DMEM containing 10% FBS at 37 °C, in an atmosphere of 5% CO_2_. Cells were passaged when they reached 80–90% confluence.

### Propagation of recombinant adenoviruses and selection of optimal infectious titres

For virus propagation, 1 μl of Ad-control or Ad-Not-siRNA viral stock solution was added to SiHa cells in the logarithmic growth phase. When the cells appeared rounded and bead-like and approximately 60% were afloat, they were collected by centrifugation, frozen in liquid nitrogen for 15 min, then thawed in a 37 °C water bath, and vortexed for 1 min. The freeze–thaw process was repeated three times, after which the cells were centrifuged at 11,950 *g* for 5 min at 4 °C. The virus-containing supernatants were collected and added to HEK293 cells for repeated infection. The recombinant adenoviruses were repeatedly propagated using the above-described procedure until high titres were obtained.

HEK293 cells were seeded in 96-well plates at a density of 1 × 10^4^ cells/well. After 8 h of cell incubation, the obtained viral stock solutions were diluted to 1:10^2^–1:10^5^ and 100 μl was added to cells in triplicate wells. The number of viruses was counted after 24 h, and the virus titre was calculated according to the following formula: virus titre = number of positive cells × virus dilution factor / 0.1. The final recombinant adenovirus titres were 5 × 10^10^ plaque-forming units per microliter.

Healthy growing SiHa cells were seeded in 24-well plates and after reaching 50% confluence were infected with 0.1, 0.3, 0.5, 0.7, 0.9, or 1.1 μl of the recombinant adenoviruses in quadruplicate at the multiplicity of infection (MOI) of 5, 15, 25, 35, 45, and 55, respectively. The cells were observed at 24, 48, and 72 h, separately, and the fluorescent signal and cell growth status were recorded. The titre corresponding to an infection rate of > 70%, without affecting the cell conditions (MOI, 25), was selected as the optimal infectious titre. The remaining viruses were aliquoted and stored at − 80 °C until use.

### Recombinant adenovirus infection

SiHa cells were sub-cultured and after reaching 50–60% confluence were infected with the Ad-control and Ad-Not-siRNA adenoviruses at the optimal infectious titres. The fluorescence intensity in each group of cells was recorded after 24 h. Uninfected SiHa cells were used as a blank control group.

### Establishment of co-culture systems

SiHa cells were cultured as the following five groups (each in triplicate): monocultures of SiHa cells (SiHa group), monoculture of SiHa cells containing control sequence (Ad-control group), monoculture of *NOTCH 1*-silenced SiHa cells (Ad-Not-siRNA group), co-culture of HUVEC/SiHa cells (HUVEC/SiHa group), and co-culture of HUVEC/*NOTCH 1*-silenced SiHa cells (HUVEC/Ad-Not-SiHa group). SiHa cells and *NOTCH 1*-silenced SiHa cells in the logarithmic growth phase were seeded in 6-well plates at a concentration of 1 × 10^6^/ml in a total volume of 2.5 ml/well. In Transwell co-culture systems, 1.5 ml of HUVECs at a concentration of 1 × 10^5^/ml was added to the upper chamber. High-glucose DMEM containing 10% FBS was used as the medium for both monocultures and co-cultures. The SiHa cells in the lower chambers were collected after 48 h of incubation in all groups.

### Cell invasion assay

SiHa cells and *NOTCH 1*-silenced SiHa cells in the logarithmic growth phase were seeded at a concentration of 1 × 10^5^/ml in a total volume of 1.5 ml onto the Matrigel matrix coating the upper chambers of the Transwell systems. In the co-culture systems, 2.5 ml of HUVECs at a concentration of 1 × 10^5^/ml were added to the lower chambers. High-glucose DMEM containing 10% FBS was used as the medium for both the monocultures and the co-cultures. After 48 h of culture incubation, the Transwell chambers were removed and fixed in anhydrous methanol at − 20 °C, followed by washing with phosphate-buffered saline (PBS). Non-invading cells were gently wiped off with cotton swabs. The chambers were then stained with crystal violet for 3 min. After the wells were washed with PBS, five fields of view were randomly selected for each well, and the invaded cells were photographed and counted.

### Quantitative real-time PCR

SiHa cells from each group were collected after 48 h of incubation, and 1.5 ml of TRIzol reagent was added on ice. The cells were allowed to stand at room temperature for 5 min, followed by centrifugation at 13,000 rpm for 5 min. The supernatants were mixed with chloroform, followed by centrifugation and precipitation with isopropanol. After centrifugation, the DNA precipitates were washed with 75% ethanol, then dried and dissolved in diethyl pyrocarbonate treated water. Genomic DNA removal, reverse transcription, and qPCR amplification were performed according to the instructions for the TaKaRa kit. The amplification conditions were as follows: initial denaturation at 95 °C for 10 min, followed by 40 cycles at 95 °C, 15 s, 60 °C, 15 s, and 72 °C, 30 s. mRNA levels of the target genes were calculated using the 2^-ΔΔCt^ method.

### Gelatine zymography

Gelatine zymography was used to semi-quantitatively determine protein and activity levels of MMP-2 and MMP-9. Briefly, proteins were separated by SDS-PAGE in gels containing 1 mg/ml gelatine. The gels were then treated with 2.5% Triton X-100 for 30 min at room temperature. The zymograms were subsequently incubated overnight at 37 °C in developing buffer. The gels were stained with 0.5% Coomassie blue R-250 and destained in 10% acetic acid and 40% ethanol in dH_2_O. Image acquisition software (UVP Inc., USA) was used for densitometric analysis of lytic bands.

### Immunofluorescence assays

SiHa cells were washed with PBS in 6-well plates and fixed with 4% paraformaldehyde for 15 min at room temperature, followed by washing with PBS. Next, 0.25% Triton X-100 was added for 15 min for membrane permeabilisation, followed by incubation with a 5% bovine serum albumin blocking solution containing 0.25% Triton X-100 for 30 min. After blocking, the cells were incubated with primary MMP-2 and MMP-9 (diluted 1:500 in the blocking solution) overnight at 4 °C, followed by washing with PBS and incubation for 1 h at room temperature with DyLight 594-labelled secondary antibodies (diluted 1:2000). Finally, the cells were washed with PBS and photographed under a fluorescence microscope. The Image-Pro Plus software (Media Cybernetics, Rockville, MD, USA) was used to analyse the fluorescence.

### Western blotting

SiHa cells from each group were washed with PBS and incubated with RIPA lysis buffer on ice. The lysates were centrifuged at 13,000 rpm, and protein concentrations were determined in the supernatants. The proteins were denatured by boiling in 4× loading buffer for 5 min and stored at − 20 °C until use. Equal amounts of protein were separated by electrophoresis in 10% separation gel and 5% stacking gel and then transferred to membrane, which were blocked and incubated overnight with primary antibodies against NOTCH 1, LOX, −SNAIL1, and β-actin (all diluted 1:800). The membranes were then washed with PBS, incubated with secondary antibodies (diluted 1:2000) for 1.5 h at room temperature, washed with PBS again, and visualised with ECL reagents. X-ray films were developed and photographed. The Image-Pro Plus software was used to analyse the density of the immunoreactive bands.

### Statistical analysis

Data were analysed using the SPSS 21.0 software (IBM, Armonk, NY, USA). Multivariate analysis of variance was used for comparisons among multiple groups and the least significant difference *t* test was used for comparisons between two groups. Differences with *P* < 0.05 were considered statistically significant.

## Results

### NOTCH 1 expression in SiHa cells

**Fig. 1:**
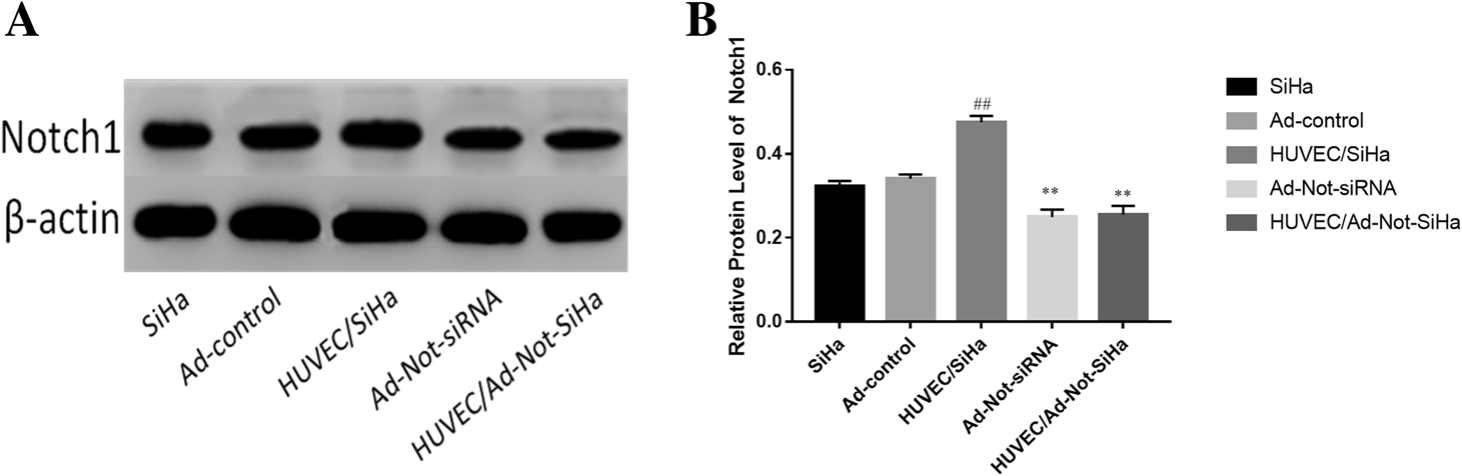
Changes in Notch l protein expression in cells. **a** Western blotting experimental strip; **b** semi-quantitative analysis of Western blotting experimental strip. Compared with the Ad-Control group, ^*##*^*P* < 0.01; Compared with the HUVEC/SiHa group, ^**^P < 0.01

The results showed that, compared with the SiHa group, there was no significant difference in Notch1 levels in SiHa cells in the Ad-control group, whereas a significant increase in Notch1 levels was observed in the SiHa cells in the HUVEC/SiHa group. Compared with the Ad-control group, the Notch1 levels were significantly lower in the Ad-Not-siRNA and HUVEC/Ad-Not-SiHa groups (Fig. 1a, b).

### SiHa cell invasiveness

**Fig. 2:**
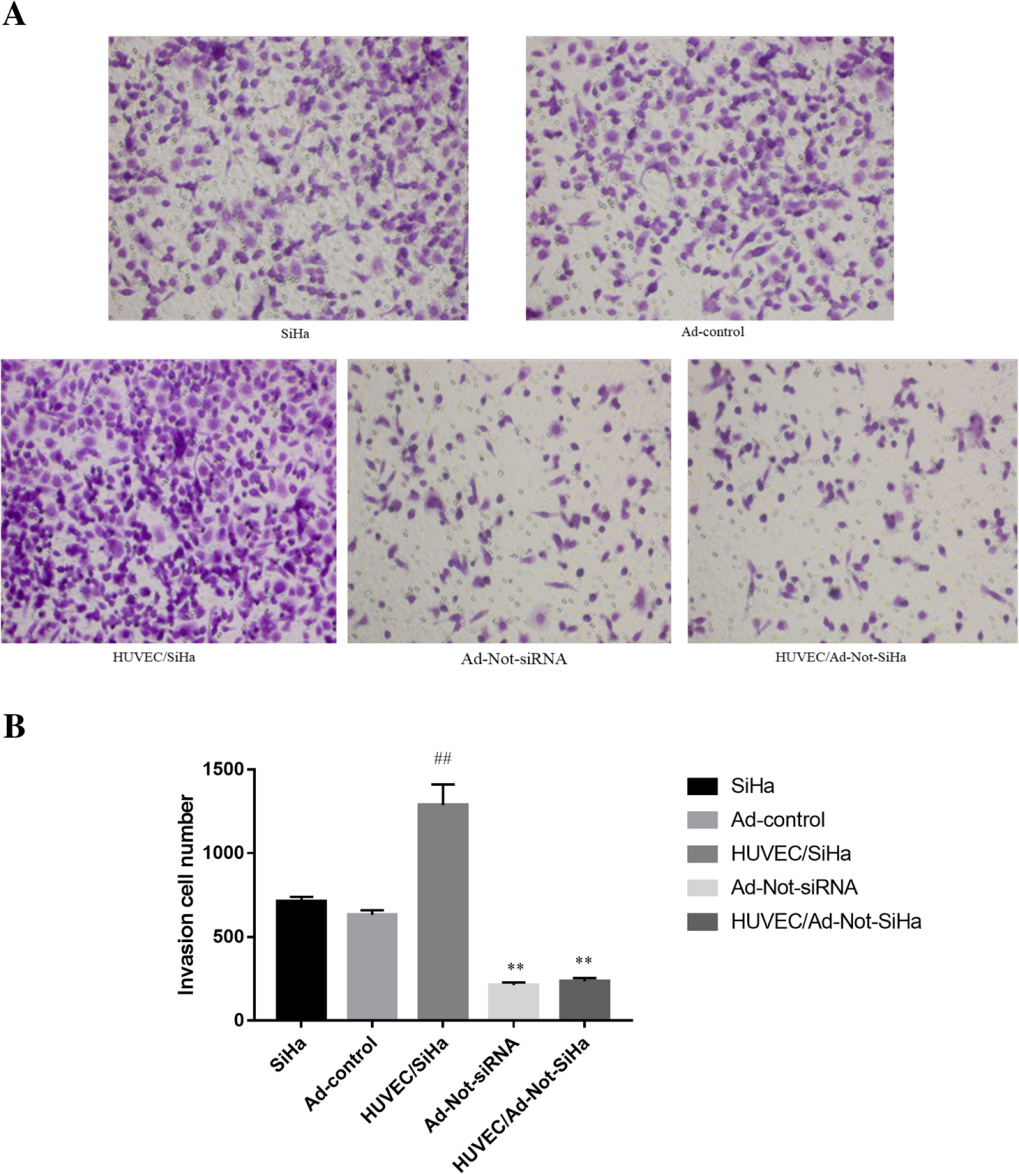
Changes in cell invasive ability. **a** Microscopic observation of cell invasion in the Transwell chamber; **b** statistical analysis of invasion. Compared with the Ad-Control group, ^*##*^*P* < 0.01; compared with the HUVEC/SiHa group, ^**^P < 0.01

The cell invasion assay showed that there was no significant difference in the invasiveness between SiHa cells without and with the Ad-control, whereas SiHa cells from the HUVEC/SiHa group showed a significantly higher invasive capacity and displayed a more mesenchymal morphology, with a spindle-like shape. Compared with that in the Ad-control group, the invasive capacity was significantly lower in SiHa cells from the Ad-Not-siRNA and HUVEC/Ad-Not-SiHa groups, both of which exhibited a more cobblestone-like cell phenotype (Fig. 2a, b).

### E-cadherin and vimentin mRNA levels in SiHa cells

**Fig. 3:**
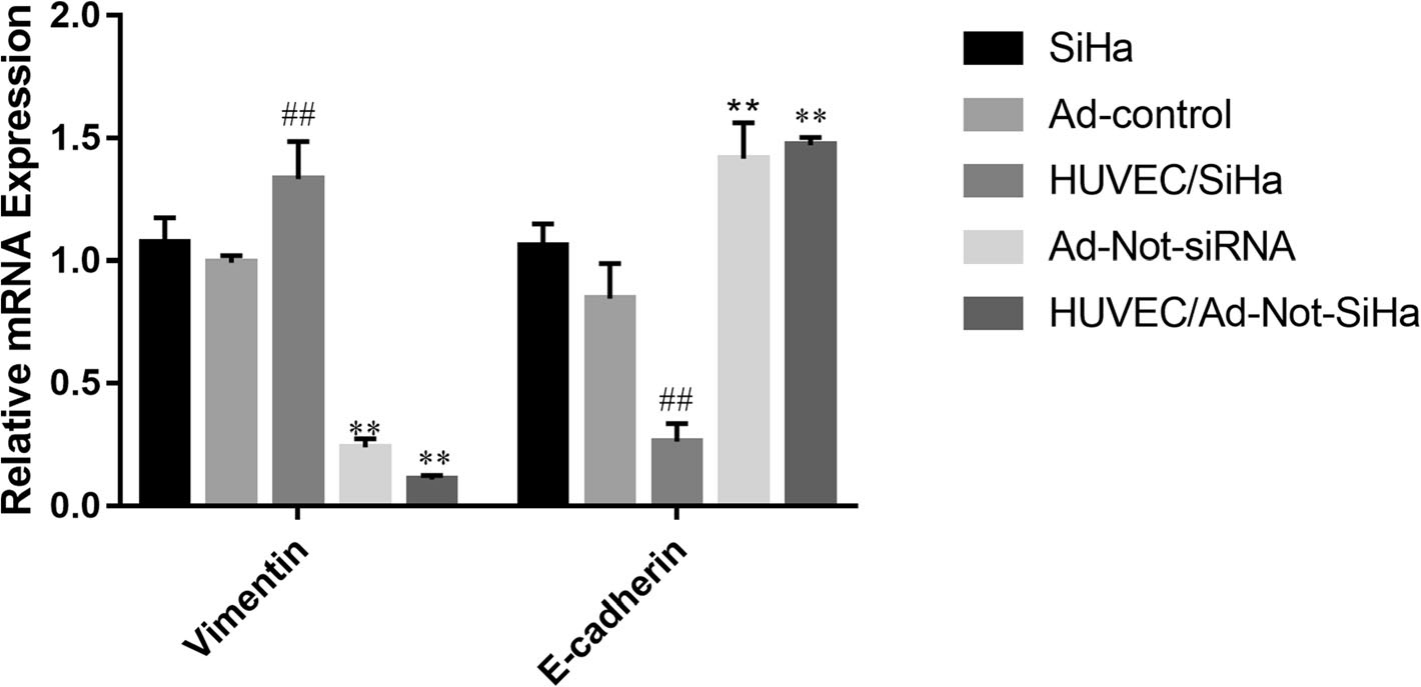
qPCR was used to detect the expression of vimentin and E-cadherin mRNA in cells. Compared with the Ad-Control group, ^*##*^*P* < 0.01; compared with the HUVEC/SiHa group, ^**^P < 0.01

qPCR showed that there were no significant differences in the mRNA levels of E-cadherin and vimentin between SiHa cells without and with the Ad-control, whereas in SiHa cells from the HUVEC/SiHa group, the mRNA level of vimentin was significantly increased, while that of E-cadherin was significantly decreased. Compared with those from the Ad-control group, SiHa cells from the Ad-Not-siRNA and HUVEC/Ad-Not-SiHa groups were showed a significant decrease in the protein mRNA levels of vimentin and a significant increase in the mRNA levels of E-cadherin (Fig. 3).

### LOX and SNAIL1 protein expression in SiHa cells

**Fig. 4:**
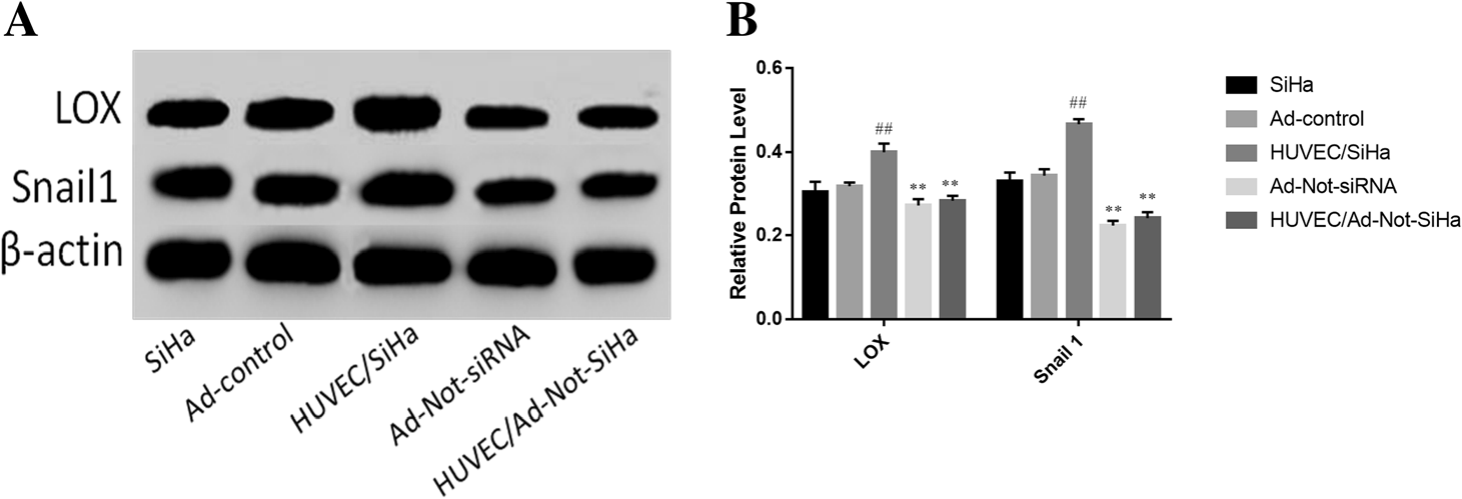
Changes in LOX and Snail1 protein expression in cells. **a** Western blotting experimental strip; **b** Western blotting experimental strip semi-quantitative analysis. Compared with the Ad-Control group, ^*##*^*P* < 0.01; compared with the HUVEC/SiHa group, ^**^P < 0.01

SNAIL1 has been previously shown to inhibit E-cadherin expression and activate vimentin expression during EMT, thereby promoting EMT. In addition, LOX has been demonstrated to stabilise SNAIL1. Therefore, we evaluated the levels of LOX and SNAIL1 proteins using western blotting. The results showed that there were no significant differences in the protein levels of LOX and SNAIL1 between SiHa cells without and with the Ad-control, whereas levels were significantly increased in SiHa cells from the HUVEC/SiHa group. Compared with those in the Ad-control group, the LOX and SNAIL1 protein levels were significantly decreased in SiHa cells from the Ad-Not-siRNA and HUVEC/Ad-Not-SiHa groups (Fig. 4a, b).

### MMP-2 and MMP-9 protein expression

**Fig. 5:**
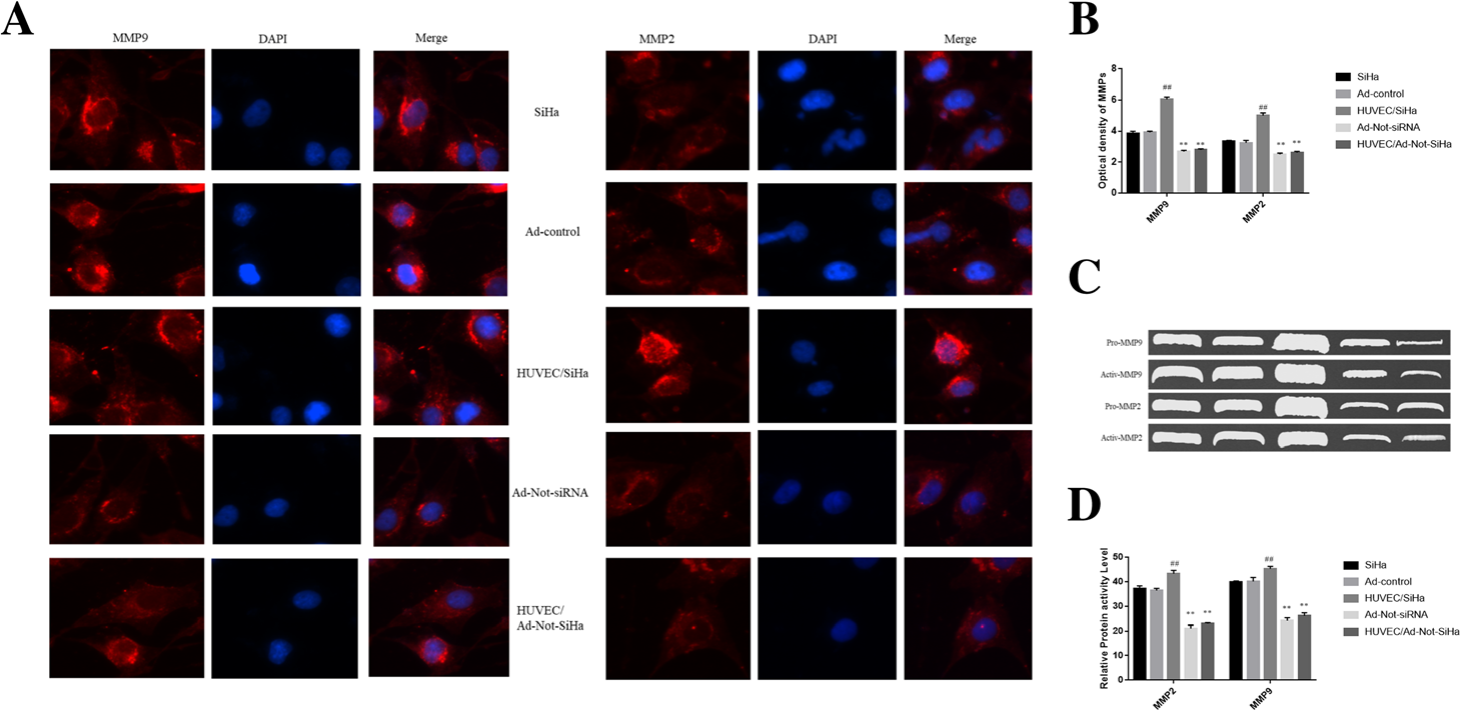
Changes of MMP-2 and MMP-9. **a** Immunofluorescence assays of MMP-2 and MMP-9; **b** Optical density of immunofluorescence; **c** Gelatin zymography – the activity of MMPs; **d** Differences analysis of MMPs’ gelatin enzyme spectrum. Compared with the Ad-Control group, ^*##*^*P* < 0.01; compared with the HUVEC/SiHa group, ^**^P < 0.01

Gelatin zymography was used to detect pro- and active forms of MMP-2 and MMP-9. Both MMP-2 and MMP-9 were expressed in all samples, and the differences between SiHa cells without and with the Ad-control were not significant. In the HUVEC/SiHa, the MMPs activities were significantly increased, and in the Ad-Not-siRNA and HUVEC/Ad-Not-siRNA groups, they were significantly reduced (Fig. 5a-d).

## Discussion

In epithelial cell-derived tumours, EMT is critical for them to acquire characteristics such as a reduced level of differentiation and increased metastatic potential. EMT is an important driver of tumour progression, and the process may be promoted by many factors. EMT is regulated at multiple levels by a regulatory network that includes tissues, cells, molecules, and the environment. At the cellular level, interactions between tumour and non-tumour cells play an important role in regulating EMT of the former. An example of such non-tumour cells is the vascular endothelial cells, which are single-layered squamous cells that line the inner surfaces of blood vessels, lymphatic vessels, and the heart, among other structures, forming the inner layer of the blood vessel wall. In tumour tissues, vascular endothelial cells interact with tumour cells to promote their EMT. Studies have found that vascular endothelial growth factor (VEGF) and insulin-like growth factor (IGF) secreted by vascular endothelial cells in head and neck squamous cell carcinoma promote EMT and metastasis of tumour cells via the VEGFR-2/HEF1/AKT/Twist1 pathway. In the present study, we found that, compared with that of SiHa cells in monoculture, the invasion capacity of SiHa cells co-cultured with HUVECs was significantly increased, suggesting that HUVECs promote the metastasis of SiHa cells. Moreover, we found that HUVECs could increase the expression of vimentin and SNAIL1 and inhibit that of E-cadherin in SiHa cells. During EMT, there is a loss of epithelial markers such as E-cadherin and cytokeratin, resulting in the loss of cell polarity and intercellular junctions. Furthermore, upregulation of mesenchymal markers, such as MMPs, vimentin, and α-smooth muscle actin, induces morphological changes in cells and enhances their ability to degrade the extracellular matrix. Overall changes in these factors cause cells to undergo EMT and promote tumour metastasis [17]. The transcription factor Snail can regulate EMT; in particular, activated Snail recognises and binds to the E-box sequence of the E-cadherin gene, which inhibits its expression and promotes the EMT of cells [18, 19]. In summary, HUVECs may promote EMT and induce metastasis of SiHa cells.

EMT may be affected by the activity of many signalling pathways. Multiple studies have found that the Notch signalling pathway is closely associated with EMT in cervical cancer. Zagouras et al. [20] showed that Notch1 was expressed in carcinoma in situ and in invasive squamous cell carcinoma of the cervix. In addition, a higher level of the NOTCH 1 receptor was detected in cervical adenocarcinoma tissues, whereas it was absent in normal cervical tissues, implicating NOTCH 1 receptors in cervical cancer. Daniel et al. [21] found that Notch1 was activated during the progression of grade III cervical intraepithelial neoplasia to cervical cancer, with intense staining of NOTCH 1 in both the cytoplasm and the nucleus. The expression of NOTCH 1 receptor gradually increased during the progression from cervical intraepithelial neoplasia to squamous cell carcinoma of the cervix, demonstrating that NOTCH 1 was highly expressed in the tissues of cervical cancer. The results of the previous study also showed that, during progression from grade III cervical intraepithelial neoplasia to micro-invasive carcinoma, the localisation of NOTCH 1 receptor shifted from the cytoplasm to the nucleus. In vitro and in vivo studies have shown that the introduction of an antisense oligonucleotide to human *NOTCH 1* into the HPV16-positive cervical cancer cell line Ca Ski can inhibit tumour cell growth and reduce the tumourigenicity of the NOTCH 1 receptor. These results indicate that NOTCH 1 is essential in the transformation of cervical epithelial cells. In cervical cancer, TGF-β can activate the NOTCH 1 receptor, induce Snail expression, inhibit E-cadherin expression, and promote EMT. We thus assessed whether HUVECs could induce the metastasis of SiHa cells through NOTCH 1 and found that silencing of *NOTCH 1* expression in SiHa cells significantly decreased the invasive capacity. In non-contact co-cultures of *NOTCH 1*-silenced SiHa cells and HUVECs, the promoting effect of HUVECs on the invasiveness of SiHa cells was lost, suggesting that HUVECs induced metastasis of SiHa cells through Notch1. Further molecular studies showed that following *NOTCH 1* knockdown, the HUVEC-induced increase in the expression of vimentin and SNAIL1 and the decrease in the expression of E-cadherin were abolished. These results demonstrate that HUVECs can promote EMT and induce metastasis of SiHa cells by activating NOTCH 1.

## Conclusions

In summary, we found that HUVECs promote metastasis of the cervical cancer cell line SiHa, which may potentially be attributed to a HUVEC-secreted protein that acts on NOTCH 1 in SiHa cells, which in turn activates the EMT in the SiHa cells. The putative protein remains to be identified in future research.

## Data Availability

The data that support the findings of this study are available from the corresponding author upon reasonable request.

## Abbreviations

DEPC: Diethyl pyrocarbonate
EMT: Epithelial-mesenchymal transition
HUVECs: Human umbilical vein endothelial cells
lncRNAs: Long noncoding RNAs
MOI: Multiplicity of infection
